# P-968. Strategies to Optimize Candidemia Management at Hospital Discharge

**DOI:** 10.1093/ofid/ofaf695.1168

**Published:** 2026-01-11

**Authors:** Joy Nash, S Lena L Kang-Birken, Fiona Asigbee, Sarah Thompson

**Affiliations:** Santa Barbara Cottage Hospital, Santa Barbara, California; Santa Barbara Cottage Hospital, University of the Pacific Thomas J Long School of Pharmacy, Santa Barbara, CA; Santa Barbara Cottage Hospital, Santa Barbara, California; Santa Barbara Cottage Hospital, Santa Barbara, California

## Abstract

**Background:**

Candidemia or invasive candidiasis (C/IC) are associated with high rates of morbidity and mortality. An empiric echinocandin is recommended by the treatment guidelines. However, the optimal time to de-escalate or transition to outpatient management has not been clearly defined due to limited data, especially among neutropenic patients, and delayed susceptibility results from send-out practice. We aimed to evaluate the utility of a C/IC transition criteria and the impact on hospital discharge and associated healthcare cost.

**Methods:**

A single center, retrospective observational study was conducted on hospitalized adult patients with documented *Candida* species growing from a sterile site culture who received an echinocandin regardless of neutropenia or intensive care unit (ICU) admission between January 1^st^, 2019 to December 31^st^, 2024. Patients with endocarditis, osteomyelitis, or meningitis were excluded. A C/IC transition criteria was developed to assess the appropriate time to transition to oral fluconazole (OF) or long acting injectable echinocandin (LAI-E) rezafungin to initiate discharge planning. The primary endpoint was the proportion of patients eligible for discharge with either OF or LAI-E. Secondary endpoints were differences in hospital length of stay (LOS) and associated cost based on transition criteria eligibility.

**Results:**

Of 107 study patients, the mean age was 63 years, 60% were male, 11% were in the ICU and 3% were neutropenic. The common comorbidities included diabetes (27%), congestive heart failure (12%) and chronic kidney disease (12%). Twenty-three patients died before eligibility could be assessed. In total, 36% met the transition criteria for LAI-E, among whom 25% were also eligible for OF. Younger age, Hispanic ethnicity, private insurance, or Medicare insurance were predictors for LAI-E qualification. Using the transition criteria for OF and LAI-E respectively, LOS would decrease by 5.4 days and 5.9 days and healthcare costs would decrease by $13,192 and $14,307 per patient.

**Conclusion:**

Optimization of I/IC treatment using a customized transition criteria and innovative outpatient method is an effective antifungal stewardship tool with potential to decrease LOS and healthcare costs.

**Disclosures:**

All Authors: No reported disclosures

